# Cutaneous Vasculitis and Recurrent Infection Caused by Deficiency in Complement Factor I

**DOI:** 10.3389/fimmu.2018.00735

**Published:** 2018-04-11

**Authors:** Sira Nanthapisal, Despina Eleftheriou, Kimberly Gilmour, Valentina Leone, Radhika Ramnath, Ebun Omoyinmi, Ying Hong, Nigel Klein, Paul A. Brogan

**Affiliations:** ^1^Infection Inflammation and Rheumatology Section, Great Ormond Street Institute of Child Health, University College London, Great Ormond Street Hospital NHS Foundation Trust, London, United Kingdom; ^2^Department of Pediatrics, Faculty of Medicine, Thammasat University, Pathumthani, Thailand; ^3^Great Ormond Street Hospital NHS Foundation Trust, London, United Kingdom; ^4^Department of Paediatric Rheumatology, Leeds Teaching Hospitals NHS Trust, Leeds, United Kingdom; ^5^Department of Histopathology, St. James University Hospital, Leeds, United Kingdom

**Keywords:** complement factor I, vasculitis, infection, complement deficiency, autoinflammation

## Abstract

Cutaneous leukocytoclastic vasculitis arises from immune complex deposition and dysregulated complement activation in small blood vessels. There are many causes, including dysregulated host response to infection, drug reactions, and various autoimmune conditions. It is increasingly recognised that some monogenic autoinflammatory diseases cause vasculitis, although genetic causes of vasculitis are extremely rare. We describe a child of consanguineous parents who presented with chronic cutaneous leukocytoclastic vasculitis, recurrent upper respiratory tract infection, and hypocomplementaemia. A homozygous p.His380Arg mutation in the complement factor I (CFI) gene *CFI* was identified as the cause, resulting in complete absence of alternative complement pathway activity, decreased classical complement activity, and low levels of serum factor I, C3, and factor H. C4 and C2 levels were normal. The same homozygous mutation and immunological defects were also identified in an asymptomatic sibling. CFI deficiency is thus now added to the growing list of monogenic causes of vasculitis and should always be considered in vasculitis patients found to have persistently low levels of C3 with normal C4.

## Background

Leukocytoclastic vasculitis (LV) is the term commonly used to describe histopathological findings of small vessel vasculitis characterized by leukocytoclasis and the presence of nuclear debris from infiltrating neutrophils. LV commonly affects the skin (cutaneous LV, CLV), and typically presents as palpable purpura of the lower extremities (1, 2). Circulating immune complexes, sometimes triggered by drugs or infections, are deposited within small blood vessels and incite an inflammatory response which includes activation of complement, resulting in small vessel vasculitis (3–5). CLV should not be regarded as a specific diagnosis in itself, since there are many underlying causes, including the primary systemic vasculitides [such as the antineutrophil cytoplasmic antibody (ANCA) associated vasculitides, IgA vasculitis, and many others]; drug reactions and other causes of hypersensitivity vasculitis; and various autoimmune connective tissue diseases (1, 6, 7). Increasingly, however, it is recognised that vasculitis (including LV) may be a presenting feature of an ever expanding list of monogenic autoinflammatory diseases, including: deficiency of adenosine deaminase type 2 (8, 9); STING-associated vasculitis of infancy (10); and other emerging genetic immunodysregulatory diseases (11); monogenic defects in complement (12, 13); and miscellaneous even rarer genetic syndromes (14). Most cases of CLV are, however, ultimately deemed idiopathic (1, 15).

The complement system is a complex and evolutionarily ancient component of the innate immune system. It is the first line of defense against invading organisms or foreign proteins, and enhances antibody function and phagocytosis (16). Activation of classical, alternative, or lectin complement pathways are documented in several vasculitides including urticarial vasculitis (17, 18), and ANCA-associated vasculitis (19). The complement system is composed of more than 30 proteins that function as complement cascade components, or system regulators (20). Genetic mutations of [including loss-of-function or gain-of-function (GOF) mutations], or autoantibodies directed to specific complement proteins can result in a broad spectrum of severe immunological sequelae that includes: recurrent bacterial infections, autoimmunity, paroxysmal nocturnal haemoglobinuria, glomerulonephritis, atypical haemolytic uremic syndrome (aHUS; typically associated with mutation in factor H, heterozygous mutations in: factor I, C3 or factor B; and mutations leading to membrane co-factor protein deficiency), and hereditary angioedema (associated with C1 esterase inhibitor deficiency) (20–22). Herein, we describe a child with recessive mutation in complement factor I (*CFI*) with deficiency of CFI causing chronic CLV and recurrent bacterial infections.

## Case Presentation

**Figure 1:**
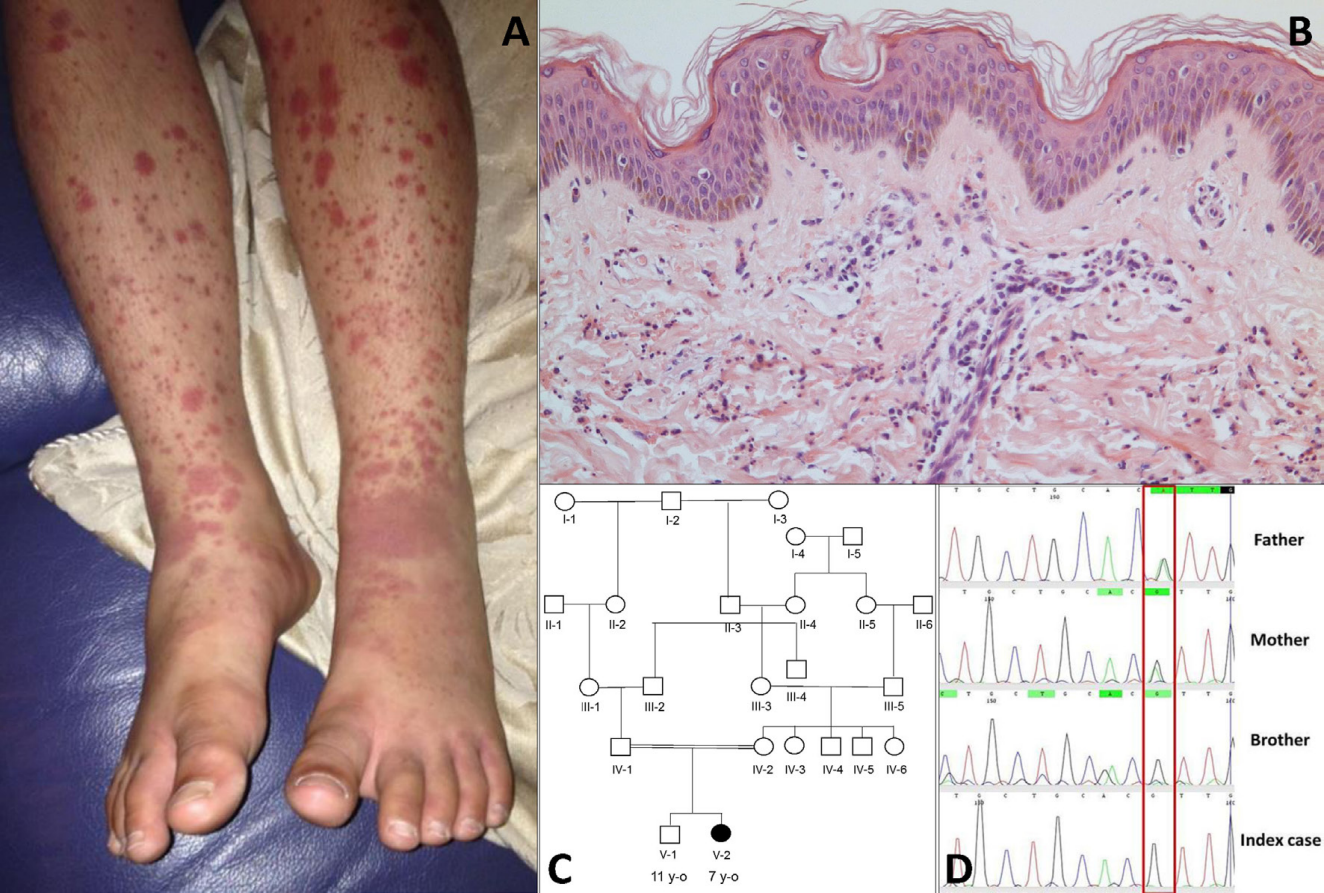
**(A)** Cutaneous vasculitis over both lower extremities of the index case [V-2; **(C)**]. **(B)** Skin biopsy of the lesion showing a dermal perivascular and interstitial infiltrate of predominantly neutrophils and nuclear dust which is compatible with leukocytoclastic vasculitis (haematoxylin & eosin stain, high magnification). **(C)** Family pedigree. **(D)** Sanger sequencing confirmed a homozygous G/G mutation (single black line) at position 1,139 of *complement factor I* gene in the index case and the asymptomatic brother; a heterozygous A/G state (Green/black line) was confirmed in both parents.

The index case was a female born to consanguineous Pakistani parents. She was well during the first year of life and tolerated her primary immunisations including BCG. From the second year of life she developed recurrent episodes of purulent otitis media (4 to 6 episodes per year) requiring multiple courses of antibiotics; although bacterial otitis was inconsistently documented, *Staphylococcus aureus* was detected on at least one occasion. She developed hearing loss secondary to these episodes, and required hearing aids. At the age of 3 years she developed a recurrent vasculitic rash affecting her arms, legs, and lower abdomen with palpable purpura (Figure 1A) this was associated with abdominal pain and arthralgia of the knees suggestive of a diagnosis of chronic IgA vasculitis (previously referred to as Henoch Schönlein purpura). Renal function and blood pressure were normal, and there was no evidence of proteinuria. Skin biopsy at that stage revealed a dermal perivascular (mainly surrounding capillaries) and interstitial infiltrate composed predominantly of neutrophils and nuclear dust (Figure 1B). Direct immunofluorescence for IgG, IgA, IgM, and C3 was negative. A second skin biopsy revealed the same picture. At the age of 4 years she developed pneumonia complicated by empyema that required drainage and intravenous antibiotics, although no organism was cultured during that episode. Between the age of 5 and 6 years she developed two further episodes of lower respiratory tract infection requiring antibiotics, and had persistent cutaneous vasculitis despite intermittent courses of oral prednisolone (1–2 mg/kg/day), and daily azathioprine (1–3 mg/kg). Therefore, at the age of 7 years she was referred to Great Ormond Street Hospital NHS Foundation Trust (GOSH) for further investigation of persistent cutaneous vasculitis and recurrent infection.

The family history (Figure 1C) revealed that parents were first cousins and were clinically well; her brother (aged 11 years) was also fit and well. Moreover, after specific enquiry, there was no history of recurrent or severe infections in the brother (V-1), or in either parent. There was also no confirmed family history of autoimmunity or immunodeficiency. On review of systems of the index case, there was no current history of prolonged or periodic fevers, arthralgia, or other systemic features. Physical examination demonstrated an injected left middle ear with effusion; and vasculitic rash affecting the lower limbs (Figure 1A). Blood pressure was normal, and there was no significant proteinuria or other organ specific involvement with vasculitis.

Laboratory investigations (Table S1 in Supplementary Material) revealed a high erythrocyte sedimentation rate of 60 mm/hr, and normal C-reactive protein of 6 mg/L [reference range (RR) <20]. All full blood count parameters were within normal limits. Rheumatoid factor was positive (59 international units/ml; RR 0–20). Other autoantibodies (antinuclear antibody; ANCA, including anti-proteinase 3 and anti-myeloperoxidase; anti-double stranded DNA; anticardiolipin; and against extractable nuclear antigens) were all negative. Notably, she had a persistently low serum C3 of 0.22 g/L (RR 0.75–1.65 g/L) with normal C4 of 0.36 g/L (RR 0.1–0.54 g/L) and decreased function of alternative (0% of healthy control) and classical (5% of healthy control) complement pathways. Low C3, normal C4, absent alternative complement pathway activity, and markedly decreased classical complement pathway activity persisted dur-ing follow up (measured on four separate occasions over 2 years), and prompted more detailed scrutiny of the complement pathway.

### Work Up for Suspected Complement Deficiency

Table 1 summarizes the genotype and results of complement studies. All experimental work was performed with ethical approval (ethics number 08H071382) with written informed consent from all adult participants, and parental consent, and assent for children. Whole-exome sequencing (WES) was performed in the index case. C3 and C4 measurements were performed by the immunology laboratory at GOSH using a standard nephelometry assay (BN II Siemens Healthcare, UK). Classical and alternative complement assays (EuroDiagnostics, Sweden) and MBL assays were performed by enzyme-linked immunosorbent assay (ELISA). CFI and H were measured at Pathology Imperial College Healthcare, NHS, UK using ELISA (Binding Site, UK). C1q was measured by radial immunodiffusion and C1q antibodies by ELISA at the Protein Reference Unit in Sheffield, UK.

**Table 1:** Complement assays in the index case and other family members.

Case	*CFI* genotype	CFI mg/L (% healthy control)	CFH mg/L (% healthy control)	C1q mg/L (RR 50–250)	C1q autoAb U/ml (RR 0–15)	C2 mg/L (RR 10–80)	C3 nephritic factor	C3 g/L (RR 0.75–1.65)	C4 g/L (RR 0.20–0.65)	Classical complement pathway assay (RR > 40%)	Alternative complement pathway assay (RR 15–125%)	MBL ng/ml (RR > 1,300)
Index case (V-2)	Homozygous p.His380Arg	19 (36%)	219 (40%)	151	328 (positive)	18	Negative	0.20	0.31	5%	0%	3,906
Brother (V-1)	Homozygous p.His380Arg	28 (60%)	234 (41%)	NA	NA	NA	NA	0.33	0.24	16%	0%	3,302
Mother (IV-2)	Heterozygous p.His380Arg	53 (115%)	>700 (128%)	NA	NA	NA	NA	1.46	0.35	94%	105%	NA
Father (IV-1)	Heterozygous p.His380Arg	55 (135%)	>700 (127%)	NA	NA	NA	NA	1.33	0.28	53%	17%	NA

*Abnormal results shaded grey*.

*CFI, complement factor I gene; CFI, complement factor I protein; RR, reference range; U/ml, units per milliliter; autoAb, autoantibody; MBL, mannose binding lectin; NA, not available*.

Whole-exome sequencing of the proband revealed a homozygous missense mutation (c.1139A > G; p.His380Arg) in the *CFI* gene that encodes CFI, an important complement regulatory factor that downregulates complement activation by cleaving C3b and C4b (23). This p.His380Arg mutation was predicted to be deleterious by *in silico* prediction algorithms, including Polyphen-2, SIFT, or GERP++, and is highly conserved between organisms according to the PhyloP algorithm. This homozygous mutation was confirmed to be present using Sanger sequencing in the index case (Figure 1C), and has very recently been described in homozygotic state in a single case with CFI deficiency (24). There were no other class 4 or 5 genetic variants (25) detected by WES in any of the complement pathway genes (including *CFH*), or in any other gene in the index case. Sanger sequencing in other family members confirmed that both parents were heterozygous for this mutation; and that her asymptomatic brother (V-1) was also homozygous for this mutation (Figure 1D). Subsequently, detailed complement studies were undertaken in the index case and the other first-degree family members. These results are summarized in Table 1. Both the index case (V-2) and the asymptomatic brother (V-1) had markedly low CFI levels, whilst the level in the parents was not reduced compared to controls. The C3 level in the index case and the brother were also reduced, but was normal in both parents. The classical and alternative complement assays were reduced in the index case, and brother; both parents had normal complement assays for all functional assays and individual components tested.

## Discussion

**Figure 2:**
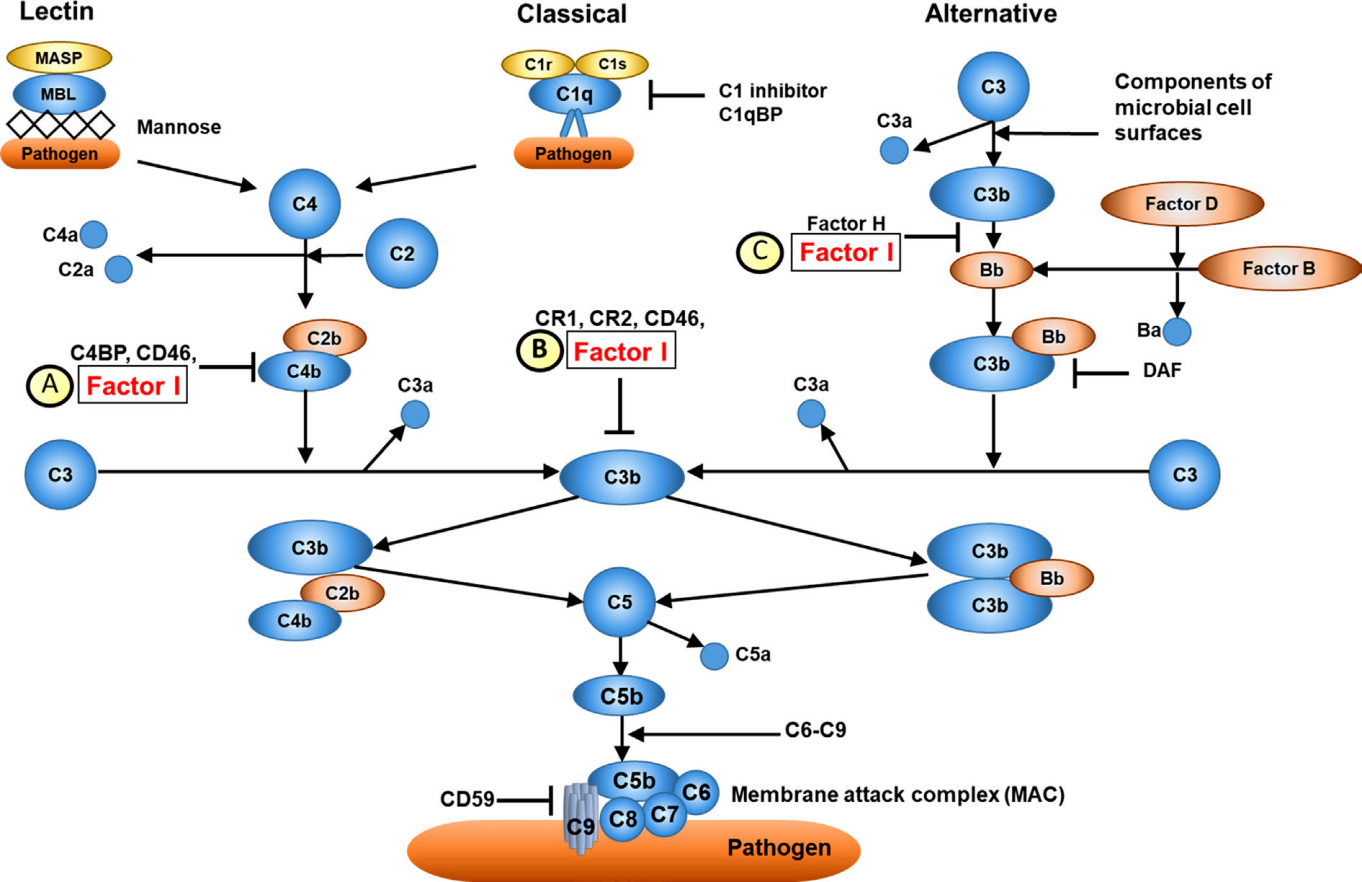
Overview of complement factor I (CFI) function. **(A)** CFI inhibits both the classical and lectin complement pathways by deactivation of C4bC2a complex. Surface-bound C4bC2a activating complex is bound by C4b-binding protein (C4BP) leading to the dissociation of C2a. CFI subsequently binds to the C4b-C4BP complex leading to the cleavage of C4b into C4c and C4d. **(B)** CFI deactivates complement receptor 1 (CR1)/CD35-bound C3b. Membrane cofactor protein (MCP/C46) binds to C3b-CR1 complex. The association of CFI results in the cleavage of C3b. **(C)** CFI inhibits the alternative complement pathway by deactivation of C3b: surface-bound C3b is cleaved into iC3B and C3d by factor H and CFI. Thus, whilst CFI is a major regulator of both the classical and alternative complement pathways, the alternative pathway is most affected, hence C3 is typically much lower than C4 in cases of CFI deficiency (see main text). Furthermore, since factor H is an important regulator of the alternative pathway, secondary factor H deficiency is observed in CFI deficiency (see main text).

*Complement factor I* is located on chromosome 4q25 (26) and encodes CFI, a serum glycoprotein produced mainly in the liver by hepatocytes. CFI is an important complement activation regulator (18); the major transcript (NM_000204) consists of 13 exons corresponding to a single heavy chain and a single light chain on the protein domain, linked by two disulphide bonds (23). The light chain harbours the serine protease (SP) domain, responsible for the catalytic activity of CFI (27). CFI regulates the activation of both the classical and alternative complement systems, but is particularly important for regulating the alternative pathway. CFI deactivates C3b and C4b by cleaving C3b into iC3b and C3d and C4b into C4c and C4d. To perform its function, CFI requires several cofactors, such as C4b-binding protein (C4BP), complement factor H, complement receptor 1 (CR1/CD35), and membrane cofactor protein (MCP/CD46); Figure 2 (21). Mono-allelic or bi-allelic mutations in *CFI* result in two different phenotypes of immunological disease. Heterozygous mutations in *CFI* are associated with aHUS, a severe disease characterized by systemic thrombotic microangiopathy (28–30). Bi-allelic loss-of-function mutations in *CFI* cause CFI deficiency, with less than 20 mutations reported in less than 50 cases worldwide (23, 31–35). Persistent alternative complement pathway activation (low C3 with normal C4) provided the clue to CFI deficiency, which was confirmed using WES in the index case. Low C3 can also be caused by bi-allelic loss-of-function C3 mutations, or mono-allelic GOF *C3* mutations (36). Our kindred did not have any mutations in *C3* (data not shown), thus excluding these important differential diagnoses.

Complement factor I deficiency results in uncontrolled amplification of C3 cleavage, which leads to consumptive C3 deficiency. As a result, patients with CFI deficiency have a defect in bacterial opsonisation leading to an increase in the susceptibility to recurrent pyogenic infections (*Haemophilus influenzae, Neisseria meningitides, and Streptococcus pneumoniae*), or non-bacterial infection. C3 consumption and dysregulation of the complement system may also lead to defects in clearance of immune complexes, and autoimmune organ injury, particularly glomerulonephritis or (more rarely) vasculitis (23, 30, 31, 34, 37, 38). The homozygous *CFI* p.His380Arg mutation we identified is a mutation in the light chain domain, which is responsible for the catalytic activity and disulfide bond subsection of CFI. Therefore, mutation of this position could affect both the catalytic cleavage function and post-translational structural modification of CFI. This amino acid is highly conserved across species, and the p.His380Arg mutation is predicted to be deleterious by all pathogenic prediction algorithms (Table S2 in Supplementary Material). Indeed, this mutation has recently been described as the cause of CFI deficiency in a 4-year-old patient (24) which taken alongside our report now irrefutably confirms its patho-genicity.

Functional complement assays of the index case (V-2) and the brother (V-1) revealed markedly decreased serum CFI, C3, CFH, and decreased activity of both the alternative and classical complement pathways. These results reflect the increased activation of both alternative and classical complement pathways. Known disease-causing mutations in *CFI* have been identified in both the light chain and heavy chains, and affect different functions and properties of CFI, including catalytic and secretory function (30). Persistently low C3 with normal C4 levels in our homozygous siblings suggests that the classical pathway was less compromised than the alternative pathway. This observation is explained by the fact that the classical pathway only requires very low concentrations (<0.1%) of its components to operate, whereas the alternative pathway requires higher concentrations (>5%) (39); hence, whilst CFI deficiency results in severe dysregulation of both alternative and classical pathways, the classical pathway is less affected and retains some function.

Dysregulation of the alternative complement pathway also explains the low factor H we observed (in the absence of any *CFH* mutations detected in this kindred) in our two patients with CFI deficiency (Table 1), since the principal function of factor H is to regulate the alternative pathway. Low levels of factor H are also described in other reports of CFI deficiency (24, 40, 41) and is secondary to consumption of the regulatory factor H presumably as a compensatory response to excessive and dysregulated alternate pathway activation.

The C1q antibodies we detected in our index case on a single occasion, in the face of normal C1q levels, are of dubious clinical significance and probably represent a non-specific epiphenomenon. In hypocomplementemic urticarial vasculitis there is a marked and selective reduction of serum C1q, in association with autoantibodies to the collagen-like region of C1q, which bind to C1q, activating the complement pathway (42, 43) Therefore, whilst we cannot completely exclude the possibility that C1q antibodies might play some secondary role to explain the vasculitic phenotype, the fact that our index case had normal C1q levels, low CFI, low C3, and low alternative pathway function which segregated perfectly with the genotype in this pedigree, and the finding of a second case of CFI recently published due to this same homozygous *CFI* mutation now irrefutably prove that it is indeed the CFI deficiency that is the main driver of the phenotype, and not primarily C1q autoantibodies.

Interestingly, the brother who also was homozygous for the p.His380Arg *CFI* mutation and had abnormal alternative and classical complement function, and low CFI, CFH, and C3 levels, is currently well, and without any history of recurrent infections. Completely asymptomatic, and mildly symptomatic individuals with confirmed CFI deficiency have also been previously described in other kindreds, but without any clear explanation for this variability in predisposition to infection (31, 44). Furthermore, reports of recurrent infections (including of the respiratory and urinary tract) have also been reported to occur with increased frequency in patients with heterozygotic *CFI* mutation and partial CFI deficiency (45). That said, there was no history of increased frequency or severity of infection in the heterozygotic parents of our kindred.

Therapeutic options for autoimmunity/autoinflammation associated with CFI deficiency are limited. Antibiotic prophylaxis and vaccination against encapsulated bacteria are pragmatic approaches to prevent infection (31, 44), which currently we have recommended for both the children described herein. It is also possible that institution of a tailored vaccination programme may improve outcomes for individuals with partial CFI deficiency as well (24, 45).

Regarding vaccination, although the production of specific antibodies against polysaccharides should not be affected by CFI deficiency, these individuals may have faster waning of antibody than healthy individuals (35), which could influence frequency of booster vaccinations. It is notable that treatment of the vasculitis with immunosuppression (azathioprine) in the index case was unsuccessful, and arguably could have contributed to the burden of infection. CFI replacement (e.g., with regular infusions of plasma) (41), would seem to have a logical approach, since it may control C3 consumption and ameliorate the vasculitic manifestations, although at the time of writing we have not yet fully explored this option. Last, we are unaware of any attempts to use liver transplantation to correct CFI deficiency, although at least in theory this might be a logical and definitive approach, albeit risky, and possibly not justifiable-based relative risks versus potential benefits in this context.

Thus, the spectrum of vasculitis caused by single gene mutations continues to expand, and CFI deficiency (or other genetic cause of alternative complement pathway dysregulation) should always be considered in patients with ANCA negative vasculitis, particularly when associated with low serum C3, but normal C4 levels.

## Ethics Statement

Written informed consent was obtained from the participants for the publication of this case report.

## Author Contributions

SN and PB: design of the work, acquisition and analysis of data, drafting and revising manuscript, providing approval for publication. DE: design of the work, revising manuscript, providing approval for publication. KG: acquisition and analysis of data, revising manuscript, providing approval for publication. VL, RR, NK: acquisition of data, revising manuscript, providing approval for publication. EO and YH: design of the work, analysis of data, revising manuscript, providing approval for publication.

## Conflict of Interest Statement

The authors declare that the research was conducted in the absence of any commercial or financial relationships that could be construed as a potential conflict of interest.
