# Supplementary material for: Cutaneous Vasculitis and Recurrent Infection Caused by Deficiency in Complement Factor I
Source: Front Immunol. 2018 Apr 11;9:735. doi: 10.3389/fimmu.2018.00735 (PMC5904195; doi:10.3389/fimmu.2018.00735)
Supplement: Supplementary file 1 [file Table_1.DOCX]

**Table S1: Laboratory investigations summary of the index case**

Summary of immunology, biochemistry and haematology investigations in the index case (V-2).

| **Laboratory investigations** | **Index case V-2 (Reference range)** |
| --- | --- |
| Haemoglobin | 127 g/L (120-160g/L) |
| Platelet count | 378x10^9^/L (150-450 x10^9^/L) |
| White blood cell count | 9.68x10^9^ /L (4.0-11 x10^9^/L) |
| Lymphocyte count | 4.30x10^9^/L (2.0-9.5 x10^9^/L) |
| Neutrophil count | 2.65x10^9^ /L (1.8-8.0 x10^9^/L) |
| Monocyte count | 0.40x10^9^ /L (0.1-0.8 x10^9^/L) |
| Immunoglobulin G | 10.7 g/L (4.9-15.6 g/L) |
| Immunoglobulin A | 2.16 g/L (0.4-0.7 g/L) |
| Immunoglobulin M | 1.52 g/L (0.4-1.9 g/L) |
| Erythrocyte sedimentation rate | 70 mm/hr (<10 mm/hr) |
| Serum amyloid A | 10.8 mg/L (<10 mg/L) |
| C-reactive protein | <5 mg/L (<20 mg/L) |
|  |  |
