# Supplementary material for: Cutaneous Vasculitis and Recurrent Infection Caused by Deficiency in Complement Factor I
Source: Front Immunol. 2018 Apr 11;9:735. doi: 10.3389/fimmu.2018.00735 (PMC5904195; doi:10.3389/fimmu.2018.00735)
Supplement: Supplementary file 2 [file Table_2.docx]

**Table S2: Pathogenic prediction score of p.His380Arg mutation in *CFI***

| **Method** | **Deleterious threshold** | **Score** | **Prediction** | **Comment** |
| --- | --- | --- | --- | --- |
| **Polyphen-2** | >0.5 | 1 | Deleterious | Predicts functional significance of amino acid substitution based on amino acid conservation and crystal structure |
| **SIFT** | >0.95 | 1 | Deleterious | Based on sequence homology and physiochemical similarity between amino acid |
| **LRT** | >0.9999 | 1 | Deleterious | Identifies if mutation disrupts conserved amino acid using multiple sequence alignment from vertebrate species. |
| **MutationTaster** | >0.5 | 1 | Deleterious | Evaluates disease causing potential of alteration to DNA sequence based on physiochemical property |
| **GERP++** | >4.4 | 5.71 | NA | Measures base conservation based on ‘rejected substitutions’ of AA to identify constrained elements which may be damaging if replaced. |
| **PhyloP** | >1.6 | 6.558 | Conserved | Calculates basewise conservation scores from alignment of 46 vertebrate species |
